# The Time-Course of Ultrarapid Categorization: The Influence of Scene Congruency and Top-Down Processing

**DOI:** 10.1177/2041669516673384

**Published:** 2016-10-19

**Authors:** Steven Vanmarcke, Filip Calders, Johan Wagemans

**Affiliations:** Brain and Cognition, University of Leuven, Belgium

**Keywords:** ultrarapid categorization, rapid gist perception, individual differences, scene congruency, object perception, psychophysical research, recurrent processing

## Abstract

Although categorization can take place at different levels of abstraction, classic studies on semantic labeling identified the basic level, for example, dog, as entry point for categorization. Ultrarapid categorization tasks have contradicted these findings, indicating that participants are faster at detecting superordinate-level information, for example, animal, in a complex visual image. We argue that both seemingly contradictive findings can be reconciled within the framework of parallel distributed processing and its successor Leabra (Local, Error-driven and Associative, Biologically Realistic Algorithm). The current study aimed at verifying this prediction in an ultrarapid categorization task with a dynamically changing presentation time (PT) for each briefly presented object, followed by a perceptual mask. Furthermore, we manipulated two defining task variables: level of categorization (basic vs. superordinate categorization) and object presentation mode (object-in-isolation vs. object-in-context). In contradiction with previous ultrarapid categorization research, focusing on reaction time, we used accuracy as our main dependent variable. Results indicated a consistent superordinate processing advantage, coinciding with an overall improvement in performance with longer PT and a significantly more accurate detection of objects in isolation, compared with objects in context, at lower stimulus PT. This contextual disadvantage disappeared when PT increased, indicating that figure-ground separation with recurrent processing is vital for meaningful contextual processing to occur.

## Introduction

To understand our visual surroundings, we need to be able to categorize the complex visual input as efficiently as possible. A semantic category can be defined as a group of two or more objects with different attributes, properties, or qualities, which are treated similarly with regard to their meaning. Within the hierarchical organization of semantic information, categorization can take place at different levels of abstraction (Rosch, Mervis, Gray, Johnson, & Boyes-Braem, 1976). The same object or scene can be categorized at a more general, superordinate level and at a less general, basic level of abstraction. Rosch et al. (1976) defined the basic object level as the level of categorization at which the categories can mirror the structure of attributes perceived in the world by sharing a common shape.

### Basic Versus Superordinate Advantage

In this classic study by Rosch et al. (1976), the basic level was identified as entry point for visual information and categorization (e.g., “dog” rather than “animal” or “golden retriever”) in a free naming task. If participants were allowed to use this level, they responded faster compared with when they used the superordinate-level categorization (Mervis & Rosch, 1981). This led to the belief that we can only label an object at a superordinate level, for example, animal, if we already know it at a basic level, for example, dog (Jolicoeur, Gluck, & Kosslyn, 1984). This idea was based on the theory that object processing occurs in a hierarchical system where the entry point or gateway was defined at the basic object level. If true, a basic-level categorization advantage should occur in every experimental design. Importantly, all experiments claiming this initial basic-level advantage used conscious (verbal) semantic labeling tasks without any time constraints (Macé, Joubert, Nespoulous, & Fabre-Thorpe, 2009; Vanmarcke & Wagemans, 2015). More recent evidence suggests, however, that learning the superordinate level does not necessarily involve basic object level knowledge. Mandler and McDonough (1993, 1998, 2000) used imitation techniques to show that very young children, when they did not label but interacted with objects to imitate a story, clearly understood and manipulated the world on a superordinate level. Even infants between 9 and 14 months old seemed to grasp superordinate concepts like animals and vehicles. This convinced the authors that the building of a conceptual system starts at a superordinate level and that children only learn to differentiate between objects on a basic level in a later stage. For example, children first learn that animals need to drink water, whereas inanimate objects do not. Later on, they learn to link the conceptual knowledge of “barking” to dogs. This does not mean however that children or infants do not see the difference between the objects at a basic level. When infants imitated a story and had to choose between different animals, they preferred the same animal as in the example shown by the experimenter. Nonetheless, children seemed to build their semantic knowledge based on a more abstract level (Mandler & McDonough, 2000). So, even before they started to use language, infants categorized on the superordinate level without clustering different basic levels together. Such a learning process seems to undermine the idea that the superordinate level is purely an abstraction of the basic level.

Interestingly, ultrarapid go/no-go categorization tasks (Macé et al., 2009; Thorpe, Fize, & Marlot, 1996; Wu, Crouzet, Thorpe, & Fabre-Thorpe, 2015) showed that the basic object level is not necessarily the semantic information activated fastest during visual processing. In such ultrarapid go/no-go categorization, a naturalistic image is briefly (20 ms) presented and participants are asked to indicate whether a predefined basic (e.g., dog) or superordinate (e.g., animal) object class is present in the display. Research indicated that people were able to do this nearly perfectly (Thorpe et al., 1996) and that participants were consistently faster at detecting an object at the superordinate level, in comparison with detecting an object at the basic level (Macé et al., 2009; Praβ, Grimsen, König, & Fahle, 2014). Similar observations (e.g., Kadar & Ben-Shahar, 2012; Rousselet, Joubert, & Fabre-Thorpe, 2005) were made for scene gist categorization in which participants have to judge the broad semantic category of the presented scene picture (e.g., “forest” or “desert”): People were faster at distinguishing natural or manmade (superordinate scene level) than sea or mountain (basic scene level). These findings were further supported by the observation that participants, when choosing between a target and a distracter image by making a saccade toward the target, needed more information when making an eye movement toward a basic object level (Wu et al., 2015). This would suggest that the visual system rapidly accesses coarse level more abstract representations of an object or scene first, before activating more fine-grained representations corresponding to a smaller category. This consistent behavioral effect in ultrarapid categorization tasks is denoted as the superordinate advantage and seems robust for increased presentation time (PT) of the stimuli (Poncet & Fabre-Thorpe, 2014). Furthermore, recent findings suggest that this perceptual categorization of rapidly presented information is also influenced by the chosen experimental trial context (Mack & Palmeri, 2015; Palmeri & Mack, 2015). Specifically, the superordinate advantage disappeared when a randomized target category design was used, in which superordinate- or basic-level categorization always changed after a few consecutive trials (e.g., maximum of four repetitions). This would suggest that the dynamics of object categorization is flexible and requires a blocked trial design, focusing specifically on either superordinate- or basic-level categories over a long series of trials, in order to observe a superordinate processing advantage in ultrarapid categorization. The current study aimed to replicate and extend these previous findings on semantic categorization by systematically manipulating the time-dependent task properties of ultrarapid categorization, within a blocked experimental trial design, and by providing an overarching theoretical framework which includes time course and task dependency.

### Theoretical Framework

McClelland and Rogers (2003) were the first to apply the parallel distributed processing (PDP) framework to hierarchical semantic categorization. In this framework, semantic processing uses the propagation of activation among simple neuron-like processing units, forming a bottom-up processing network. Initially, the connection weights between the processing units within this neural network remain uninformative. During the learning process, the weights change slowly, gradually reducing errors, and becoming informative about how the activation of units in one level of representation determines the activation at another level of representation (McClelland & Rogers, 2003; Rogers & McClelland, 2004). Later, Leabra (Local, Error-driven and Associative, Biologically Realistic Algorithm), the successor of PDP, incorporated more biologically realistic mechanisms (Aisa, Mingus, & O’Reilly, 2008). This led to the development of LVis (Leabra Vision; Wyatte, Herd, Mingus, & O’Reilly, 2012). This computational model of visual processing is capable of identifying stand-alone objects and labeling them at the basic object level (O’Reilly, Wyatte, Herd, Mingus, & Jilk, 2013). Other neural network simulations with Leabra demonstrated the critical role inhibition plays in lexical selection (Snyder et al., 2010) . In contradiction to strictly bottom-up models of object recognition, the LVis model uses recurrent processing as a key feature to correctly identify partially occluded objects (Wyatte et al., 2012). More precisely, the neural interconnectivity within- and between-brain regions helps to create a stable and clear representation of an ill-visible object by allowing high-level visual brain areas to shape the neural activation and predictions of low-level visual areas through inhibitory and excitatory recurrent connections (O’Reilly et al., 2013). Recurrent processing also plays an important role in figure-ground segmentation (Lamme, Zipser, & Spekreijse, 2002), border ownership, and subjective surface perception (Kogo & Wagemans, 2013a, 2013b). The neural activation from the low-level visual areas needs to be linked to one distinct lexical category. In ultrarapid categorization tasks, the label of the object is predefined, allowing top-down biasing of the relevant visual features. This facilitates the predefined categorization decisions, resulting in a top-down bias favoring superordinate categorization (Bar, 2004; De Cesarei, Peverato, Mastria, & Codispoti, 2015) and translating into the observed superordinate advantage in the ultrarapid categorization literature (e.g., Rogers & Patterson, 2007; Vanmarcke & Wagemans, 2015). However, the PDP theory (Rogers & McClelland, 2004; Wyatte et al., 2012) also proposed that, after initial activation, similarity-based generalization implies strong generalization within basic categories but weak generalization between them. In this way, similarity-based generalization promotes rapid and active learning of basic-level names. For instance, the name “dog” tends to generalize strongly to items with similar representations, such as other dogs, but not to items with more distal representations, such as other kinds of animals. Because superordinate category learning benefits more slowly from this similarity-based generalization in comparison to basic-level information, this superordinate advantage can turn into a basic-level advantage over time when the task requires an active, conscious labeling of different semantic categories. This dynamic network characteristic is in agreement with the finding that people are faster to confidently name and verify category membership verbally at the basic level when no time constraints are in place (Rosch et al., 1976). The same reasoning applies with regard to the influence of the experimental trial context on flexibly categorizing semantic information at different levels of abstraction (Mack & Palmeri, 2015).

### Time-Dependent Task Properties of Ultrarapid Categorization

In the current study, we use the theory summarized earlier to formulate specific experimental predictions for behavioral processing in masked ultrarapid basic- and superordinate-level categorization in a blocked experimental trial design. In contradiction with previous ultrarapid categorization research, focusing on differences in participant reaction times, the current study used accuracy as its main dependent variable. More precisely, we estimated the best-fitting sigmoid function (Weibull distribution) per participant by using maximum likelihood parameter estimation (psychometric performance curve) for categorization performance by dynamically changing stimulus PT (ranging from 16 to 80 ms). After stimulus presentations, a perceptual mask was shown in order to explicitly control for the influence of the top-down biasing of the relevant visual features during categorization (Fahrenfort, Scholte, & Lamme, 2007). This was done in an ultrarapid categorization task in which we varied two defining task variables: (a) level of categorization (basic vs. superordinate categorization) and (b) object presentation mode (object-in-isolation vs. object-in-context).

Hypothesis 1: Basic versus superordinate advantage in ultrarapid categorization.

Longer PT of the ultrarapid categorization task without perceptual masking did not reverse the superordinate advantage in a blocked experimental trial context (Poncet & Fabre-Thorpe, 2014). However, previous psychophysical studies showed that masking the stimulus presentation allowed to dissociate between the bottom-up and top-down (recurrent) components of the neural response (for review, see Breitmeyer & Ogmen, 2006; Enns & Di Lollo, 2000). Previous research on ultrarapid categorization suggested that significant top-down modulation should be possible after a stimulus presentation of 40 to 60 ms (Roland, 2010; Serre, Oliva, & Poggio, 2007). To investigate the possible influence of both bottom-up and top-down categorization processes on the behavioral performance in a masked and predefined ultrarapid categorization task, we dynamically manipulated the stimulus PT within a range of 16 to 80 ms. Based on the PDP theory and Leabra models (O’Reilly et al., 2013), and in accordance with previous research on the time course of object categorization with a blocked trial design (Mack & Palmeri, 2015), we predicted participants to show a consistent superordinate accuracy advantage across all PT (Hypothesis 1). This hypothesis followed from the unambiguously predefined search goal in ultrarapid categorization, which would allow the top-down biasing of the relevant visual features for coarse (superordinate vs. basic) scene categorization (Bar, 2004; De Cesarei et al., 2015).

Hypothesis 2: Influence of object presentation mode on ultrarapid object detection.

Objects can either be presented in isolation or can be embedded within a meaningful scene context. Previous research indicated that such contextual information can facilitate object identification compared with incongruent background information (Rémy et al., 2013). More precisely, when objects are embedded in a familiar context, for example, a plane in the sky, object recognition is both faster and more accurate than when objects are presented in an incongruent context in which they are less likely to appear, for example, a bed in a forest (Fize, Cauchoix, & Fabre-Thorpe, 2011; Joubert, Fize, Rousselet, & Fabre-Thorpe, 2008). Similar findings were observed in an ultrarapid categorization paradigm without perceptual masking (Crouzet, Joubert, Thorpe, & Fabre-Thorpe, 2012). This direct influence of context on object recognition might be related to the lifelong experience of the visual system with our visual surrounding world and its efficiency at extracting visual regularities (Davenport, 2007; Rémy et al., 2013). Furthermore, electroencephalography research showed that masking stimuli interrupts figure-ground segmentation by interrupting recurrent (top-down) processing (Fahrenfort et al., 2007). In the current study, we were therefore interested whether object-congruent background information could speed up categorization before figure-ground segmentation was completed. More concretely, we were interested in how the time course of masked ultrarapid object categorization would influence the discrimination accuracy of objects, either presented in isolation or embedded within a meaningful everyday scene. Based on the Leabra theory (O’Reilly et al., 2013), we predicted that participant performance would only be influenced by the object-congruent context when longer PT in perceptually masked rapid categorization allowed top-down processing to affect response speed (Hypothesis 2). This hypothesis was based on the expected impact of meaningful contextual information on object detection, resulting from the inherent influence of relevant global scene statistics diagnostic for object categorization on the identification of salient objects (Bar, 2004; De Cesarei et al., 2015).

## Materials and Methods

### Participants

A group of 140 participants (20 men and 120 women) was tested, with a median age of 18 (*SD* = 3.57; [min, max] age = [17, 43]; interquartile range [IQR] = 1). All participants were first-year psychology students at the University of Leuven (KU Leuven). They received course credits for participation. Participants who did not follow the task instructions or did not complete the task as requested were deleted from the data set before onset of the actual analysis. The final participant set therefore contained exactly 136 participants (20 men and 116 women), with a median age of 18 (*SD* = 1.66; [min, max] years old = [17, 29]; IQR = 1). The study was conducted in line with the ethical principles regarding research with human participants as specified in The Code of Ethics of the World Medical Association (Declaration of Helsinki). The study was approved by the Ethical Committee of the Faculty of Psychology and Educational Sciences (EC FPPW) of the University of Leuven (KU Leuven), and the participants provided written informed consent before starting the experiment.

### Computer Task

This section provides an overview of the ultrarapid categorization task completed by all participants. Participants were asked to take a comfortable position before the computer screen (at about 57 cm of the computer display) and placed both hands on the keyboard (spacebar) in front of the computer monitor (resolution: 1920 × 1080; refresh rate: 60 Hz; type: DellP2211H). The experiment was conducted using the open-source software library PsychoPy, which is written in Python (Peirce, 2008).

#### Design

**Figure 1.**
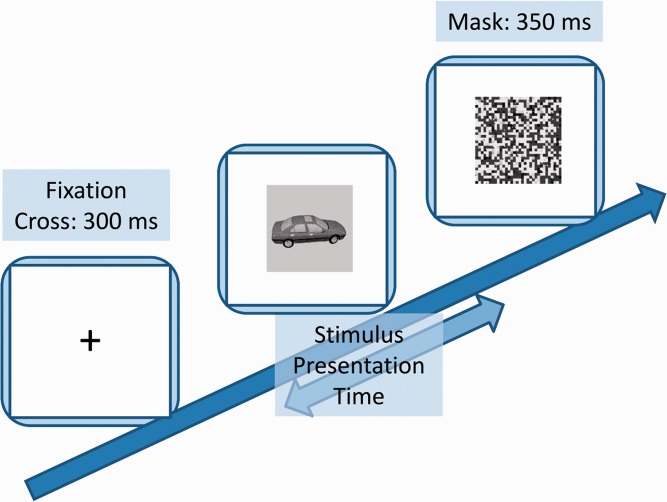
A graphical overview of the trial design of the ultrarapid categorization task.

**Figure 2.**
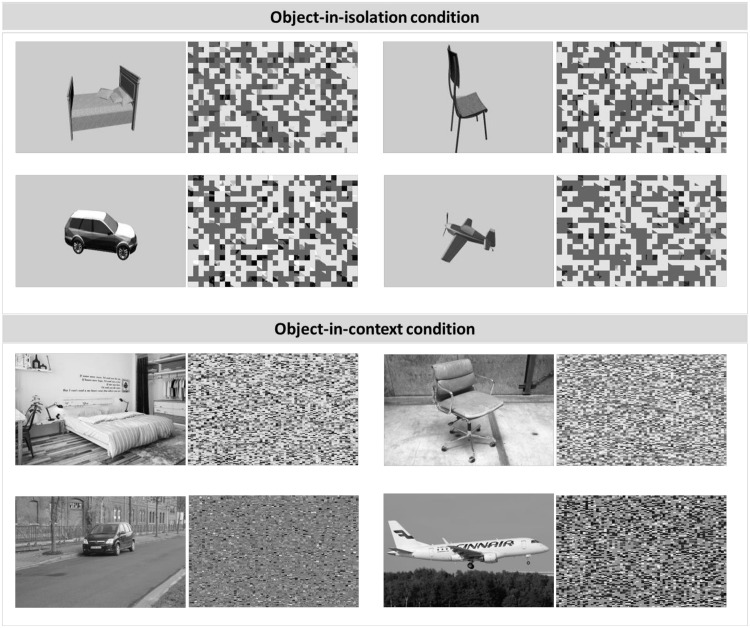
A general overview of the type of images used within the ultrarapid categorization task for both the (a) object-in-isolation and the (b) object-in-context condition. The complete picture set is made available online on http://www.gestaltrevision.be/en/resources/supplementary-material/76-resources/supplementary-material/826

**Figure 3.**
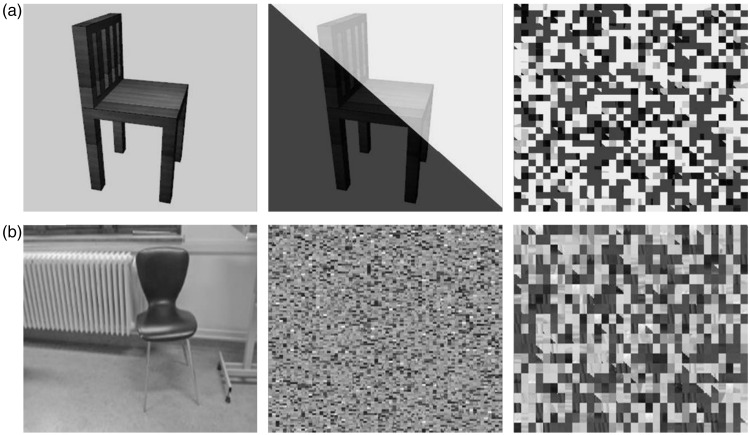
(a) To avoid a strong influence of the gray image background in the object-in-isolation condition masks, we first imposed a diagonal black-white watermark (50% transparency) before scrambling the images. (b) The first panel exemplifies one of the stimuli (e.g., a chair) used in the object-in-context condition, and the second panel shows the mask as it was made in the main experiment. The third and last panels show how the perceptual mask was constructed in the control experiment, using a black-white watermark (50% transparency), similar to the perceptual mask in the object-in-isolation condition of the main experiment.

The ultrarapid categorization task (Figure 1) took about 30 minutes, and all instructions were projected on the computer screen. Every trial started with a fixation cross (300 ms). Then the stimulus was presented for a variable duration (see later). After the stimulus presentation, a perceptual mask was shown (350 ms). The mask was computed by dividing each image into pixel-squares (2 by 2 pixels per square) and then randomly scrambling these stimulus elements (per image). Such a scrambled version was created for each stimulus in the object-in-context condition, as exemplified in Figure 2(b). To avoid a strong influence of the gray image background in the object-in-isolation condition masks, we first imposed a diagonal black-white watermark (50% transparency) before scrambling the images. This is exemplified in Figure 3(a). Importantly, rigorous pilot testing on an independent sample of naïve participants, several weeks before the main experiment, indicated that these masks, in both conditions, made it impossible (chance-level performance) to correctly categorize the stimuli when the PT was 16 ms (or less) and stimulus presentation was masked (chance-level performance). Furthermore, in the main experiment, the stimulus PT in the ultrarapid categorization task depended on subject performance. Performance was calculated every 10 stimuli and PT decreased (or increased) with performance above (or below) 75% with 16 ms. The experiment started with two alternating PT: one initialized at 16 ms, a second at 80 ms. Subjects who did not reach 75% performance at a PT of 80 ms during the entire task were assumed to be inattentive or not understanding the task. Their results were discarded before the actual data analysis began. In general, information on the following stimulus PT was collected: 16, 32, 48, 64, and 80 ms. Subjects had a 1000-ms response window and when the target was not presented, the subject had to wait until the trial ended. After the response, a new trial started about 200 ms later. In the practice trials, visual feedback was given. The word “correct” flashed in green when a correct answer was given. When the answer was incorrect, the word “wrong” flashed in red. Practice trials ended after six correct answers, and PT during these trials was always 80 ms. Analysis indicated that all subjects (except those discarded due to inattentiveness) had a high performance in the practice trials, indicating that the task was well understood. The practice trials were followed by two test sessions of 10 minutes, one test session on basic and one on superordinate object level. Between the test sessions, a break of 1 minute was given. After the task, a short debriefing followed and participants were randomly divided into different conditions.

#### Stimuli

Every participant was randomly assigned to one of two conditions (Figure 2). In the object-in-isolation condition, stand-alone objects with a gray background were used, while in the object-in-context condition, the object was embedded in a scene. For the object-in-isolation condition, we used 480 gray-scaled images (320 × 214 pixels) from the CU3D-100 dataset (O’Reilly et al., 2013). The object could be everywhere in the image but was always in the foreground and fully visible. In both the superordinate and the basic-level test session of the object-in-isolation condition, the stimuli were randomly chosen from this set of 480 gray-scaled pictures: 50% of the selected stimuli were used as targets, 50% were used as nontargets. Every stimulus was shown only once or twice. In line with previous research (e.g., Macé et al., 2009; Praβ et al., 2014), targets and nontargets of the same level of categorization were used in each stimulus category: at superordinate- and basic-level object categorization. To make this more explicit: (a) in the furniture (vehicle) category, vehicle (furniture) stimuli were used as nontargets and (b) in the bed (chair or plane or dog) category, chair, plane, and dog (bed), stimuli were used as nontargets. In each of the different image categories, a wide variety of possible scenes were selected. For the object-in-context condition, a total set of 480 gray-scaled images (320 × 214 pixels) were used for this task. These scenes were selected (by unanimous consensus between several lab members including the first two authors) on the Internet and taken with a 1NIKKOR camera. The same principles as in the objects-in-isolation condition were used to create the sets of target and nontarget stimuli. To avoid low-level confounds eliciting behavioral differences between stimulus categories and conditions (Wichmann, Braun, & Gegenfurtner, 2006), each of the selected images was set to the same global luminance and root mean square contrast (corresponding to a luminance distribution, within the gray-scale spectrum, with a mean of [110] and a standard deviation of [25.00]) by computing the average luminance and root mean square contrast across all images. The mean luminance of the images on the screen was 10 to 20 cd/m^2^.

#### Task instructions

Every participant was randomly assigned to either the object-in-context or the object-in-isolation condition. In each of these conditions, participants were asked to complete one test session on superordinate level and one on basic object level categorization (in a random order). Both types of test sessions started with an instruction question: (a) *Is there* a piece of furniture *in the photo*? (press spacebar), (b) … a vehicle … , (c) … bed … , (d) … chair … (e), … plane … , or (f) … car … . Sessions 1 and 2 are at the superordinate level, while Sessions 3, 4, 5, and 6 are at the basic ordinate level. While Session 1 was always performed together with 3 or 4, Session 2 was always performed together with 5 or 6. An exact overview of the number of participants (in the final sample) in each of the possible combination of test session is provided in Appendix A. Similar to previous findings in ultrarapid categorization (e.g., Macé et al., 2009), no between-subject differences in performance were observed for the different detection tasks (e.g., car, plane,…), for neither the object-in-isolation or the object-in-context condition, at the same basic or superordinate level of categorization.

#### Mask control experiment

It might be argued that the perceptual masks used in either the object-in-isolation (Figure 2(a)) or the object-in-context condition (Figure 2(b)) could have a differential influence on participant performance given that they visually differed substantially between both conditions (Figure 2(a) compared with Figure 2(b)). To test this possible confounding variable, we conducted a control experiment on an independent sample of 29 participants (13 men and 16 women), with a median age of 23 (*SD* = 8.51; [min, max] age = [18, 31]; IQR = 3). These participants were asked to perform the exact same go/no-go categorization task as in the main experiment. The only difference with the original set-up was the mask in the object-in-context condition. This control mask was a scrambled version of the presented image for which, similar to the object-in-isolation condition mask of the main experiment, we imposed a diagonal black-white watermark (50% transparency) before scrambling the images (Figure 3(b)). Furthermore, similar to the main experiment, in the mask control experiment, participants were asked to take a comfortable position before the computer screen (at about 57 cm of the computer display) and placed both hands on the keyboard (spacebar) in front of the computer monitor (resolution: 1920 × 1080; refresh rate: 60 Hz; type: DellP2211H). Furthermore, in this control experiment, but not in the main experiment, the head position of the participants was stabilized by means of a head and chin rest during testing.

### Analysis

**Figure 4.**
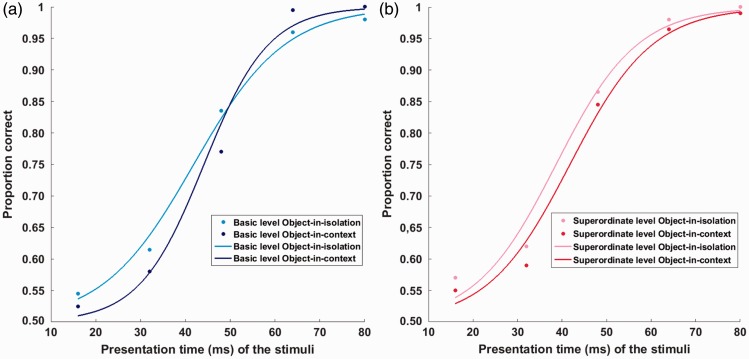
The figure provides an example of the best-fitting sigmoid functions for a single participant. In (a) the basic level object-in-isolation (light blue bar) and object-in-context (dark blue bar) conditions are presented, while in (b) the superordinate-level object-in-isolation (pink bar) and object-in-context (red bar) are provided. The main parameters of these sigmoid functions, alpha (α) and bèta (β), provided an overall estimation of the time-dependent categorization performance in each test session per participant.

For every participant separately, the accuracy data on each test session of the ultrarapid categorization task (consisting out of an average of 318 trials) were used to determine the best-fitting sigmoid function (Weibull distribution) using maximum likelihood estimation for parameter estimation (Wichmann & Hill, 2001a). This psychometric fitting was done using the Psignifit Toolbox in MATLAB R2009a (Wichmann & Hill, 2001b), with accuracy as the dependent variable (DV) and PT as the independent variable. This resulted in two separate psychometric fits per participant: one for the object-in-context and one for the object-in-isolation condition (Figure 4). The main parameters of these sigmoid functions, alpha (α) and bèta (β), provided an overall estimation of the time-dependent categorization performance in each test session per participant. We then used both α and β as the DVs in a mixed analysis of variance (ANOVA) with presentation mode (object-in-context vs. object-in-isolation condition) as a between-subjects factor and level of categorization (basic vs. superordinate) as a within-subjects factor. Participants were regarded as a random factor. Furthermore, we also used α and β separately as DVs in a general linear mixed modeling (GLMM) approach (McCullagh, 1984). Furthermore, to further pinpoint possible differences in categorization between participants, also the threshold and slope values at specific points of the individual psychometric fits (60, 75, and 90%) were taken into account. Deviance values were calculated for the regression models based on a maximum likelihood estimation (Aitkin, 1999) of all DVs on the tasks. By evaluating the drop in deviance together with the Akaike (Akaike, 1973) and Bayesian Information Criterion (Schwarz, 1978) values (for overview, see Appendix C), the final model was selected. After model selection, the individual predictive value of each selected parameter was tested using Welch’s *t* test with Satterthwaite approximation for the denominator degrees of freedom (McArdle, 1987) in the random intercepts regression analysis. Descriptive measures (e.g., age and gender) were tested as possible covariates. The mixed ANOVA analysis provided very similar results as the GLMM approach. We therefore decided only to report the GLMM outcomes and to include all other results in Appendix B. The outcomes of the GLMM were obtained by using the lme4 package (Bates, 2005) of the statistical software program R version 3.1.1 (R Core Team, 2013). The mixed ANOVA was done using IBM SPSS (Version 22).

For the psychometric function parameter α, both presentation mode (object-in-context vs. object-in-isolation condition) and level of categorization (basic vs. superordinate) were regarded as fixed effects in the final model. These observations were further refined when taking the performance of participants at specific psychometric threshold (60%, 75%, and 90%) values into account: level of categorization was a significant predictors of performance on all specified threshold levels (60%, 75%, and 90%), but presentation mode only predicted performance on the lower threshold levels (60% and 75%). Furthermore, for the psychometric function parameter β, only presentation mode (object-in-context vs. object-in-isolation condition) was withheld as a fixed effect in the final model. These observations were further refined when taking the performance of participants at specific psychometric slope (60%, 75%, and 90%) values into account: presentation mode was a significant predictor of performance for slope at all points of the curve. The Presentation Mode × Level of Categorization interaction was not significant for any of the conducted analysis. Descriptive variables such as test order, age, or gender were also not withheld as significant predictors of performance in the final models for α and β. The goodness-of-fit measures (for overview, see Appendices) for each of the parameter estimates (χ^2^) in the final GLMM model are provided in the results section. Data and an example of the analysis code are available online on http://www.gestaltrevision.be/en/resources/supplementary-material/76-resources/supplementary-material/826.

Finally, we also analyzed the data of the control experiment using the same GLMM modeling approach as in the main experiment. These findings replicated our original results and indicated that the mask type was no confounding variable in explaining the current results. We added the regression parameter estimates for the main parameters, alpha (α) and bèta (β), of the individual sigmoid maximum likelihood fits in Appendix D.

## Results

**Figure 5.**
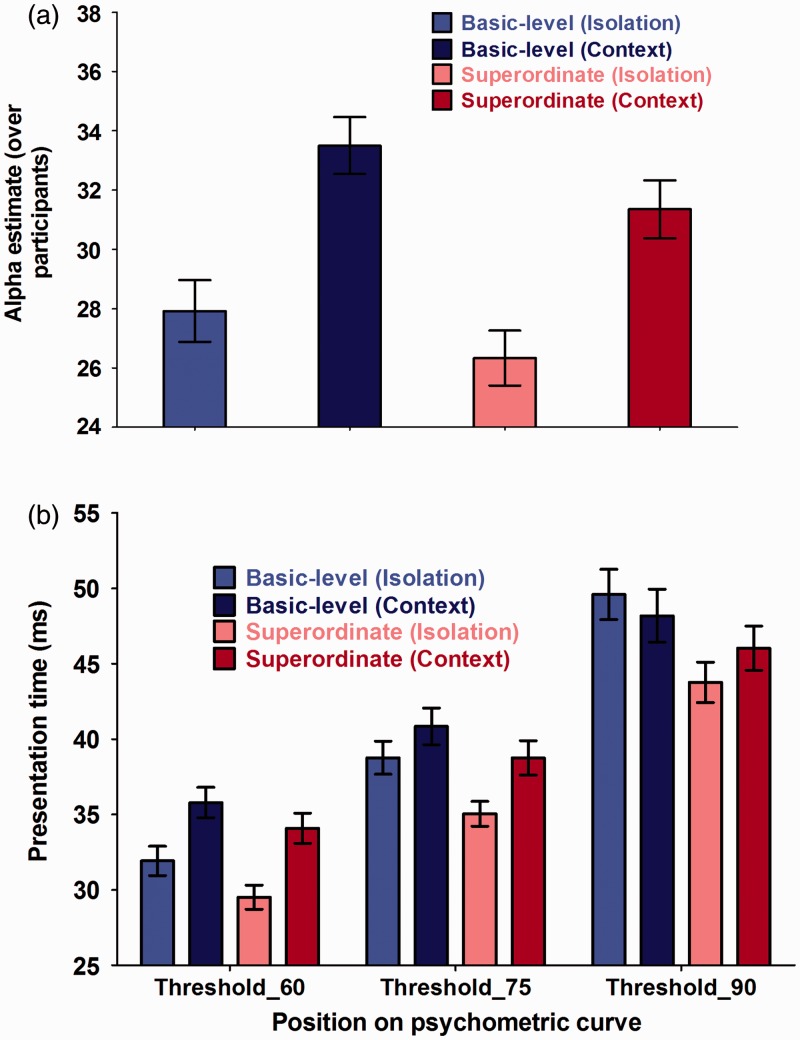
Overview of (a) average α estimates across participants and (b) average presentation time necessary to achieve an overall accuracy of 60%, 75%, or 90% correct in rapidly detecting a basic-level object-in-isolation (light blue bar), a basic-level object-in-context (dark blue bar), a superordinate-level object-in-isolation (pink bar), or a superordinate-level object-in-context (red bar). The data are represented as the mean performance across participants, with error bars depicting the standard error of the mean (*SEM*).

Hypothesis 1: Basic versus superordinate advantage in ultrarapid categorization (Figure 5).

**Figure 6.**
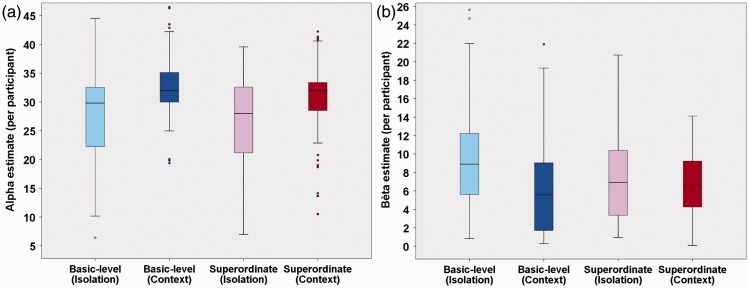
Overview of the overall distribution of the (a) α and (b) β estimates of the participants. The different boxplots represent the four different conditions: basic-level object-in-isolation (light blue bar), basic-level object-in-context (dark blue bar), superordinate-level object-in-isolation (pink bar), and superordinate-level object-in-context (red bar).

**Figure 7.**
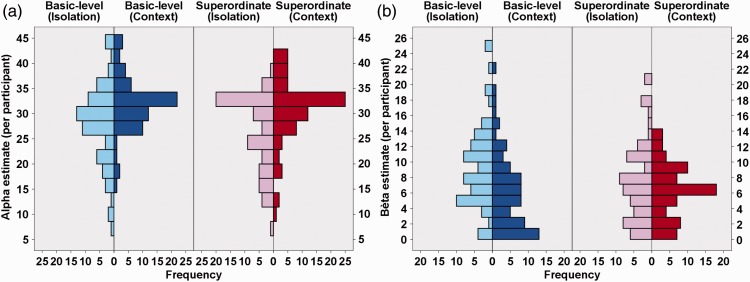
Overview of the overall distribution of the (a) α and (b) β estimates of the participants. The different side-by-side histograms represent the four different conditions: basic-level object-in-isolation (light blue bar), basic-level object-in-context (dark blue bar), superordinate-level object-in-isolation (pink bar), and superordinate-level object-in-context (red bar). While the ordinate depicts the (a) α or (b) β estimates, the frequency values are provided on the abscissa.

We found a significant main effect of the within-subjects variable level of categorization for the psychometric function parameter α (*t*(136) = −2.85; *P*(χ^2^) = 5.11 × 10^−3^). This parameter provides an estimate of the overall PT necessary for participants to correctly judge whether a predefined basic or superordinate object was presented or not. More precisely, the current results indicated that participants were generally faster at detecting superordinate (more abstract) information (e.g., vehicle) than in observing more basic (more concrete) level representations of its constituting subcategories (e.g., plane). In line with the absence of a main effect of level of categorization for the psychometric (slope) parameter β (*t*(272) = −1.32; *P*(χ^2^) = .18), this superordinate advantage in performance was found to be clearly present over all PT (60% threshold: *t*(136) = −2.83; *P*(χ^2^) = 5.41 × 10^−3^ | 75% threshold: *t*(136) = −3.17; *P*(χ^2^) = 1.86 × 10^−3^ | 90% threshold: *t*(136) =−2.60; *P*(χ^2^) = .01). Such a finding indicated that PT and categorization performance differed between basic and superordinate processing when categorization required a rapid, predefined object detection. When graphically exploring this effect further (Figures 6(a) and 7(a)), it seemed to result from a shift in threshold distribution. More precisely, the superordinate α distribution peaks earlier than the basic-level distribution due to the presence of more participants with low α values (heavier tale). This consistent processing advantage for superordinate information was in line with the PDP theory (O’Reilly et al., 2013) and our predictions (Hypothesis 1). See Table 1 for parameter estimates and 95% confidence intervals of the final models.

**Figure 8.**
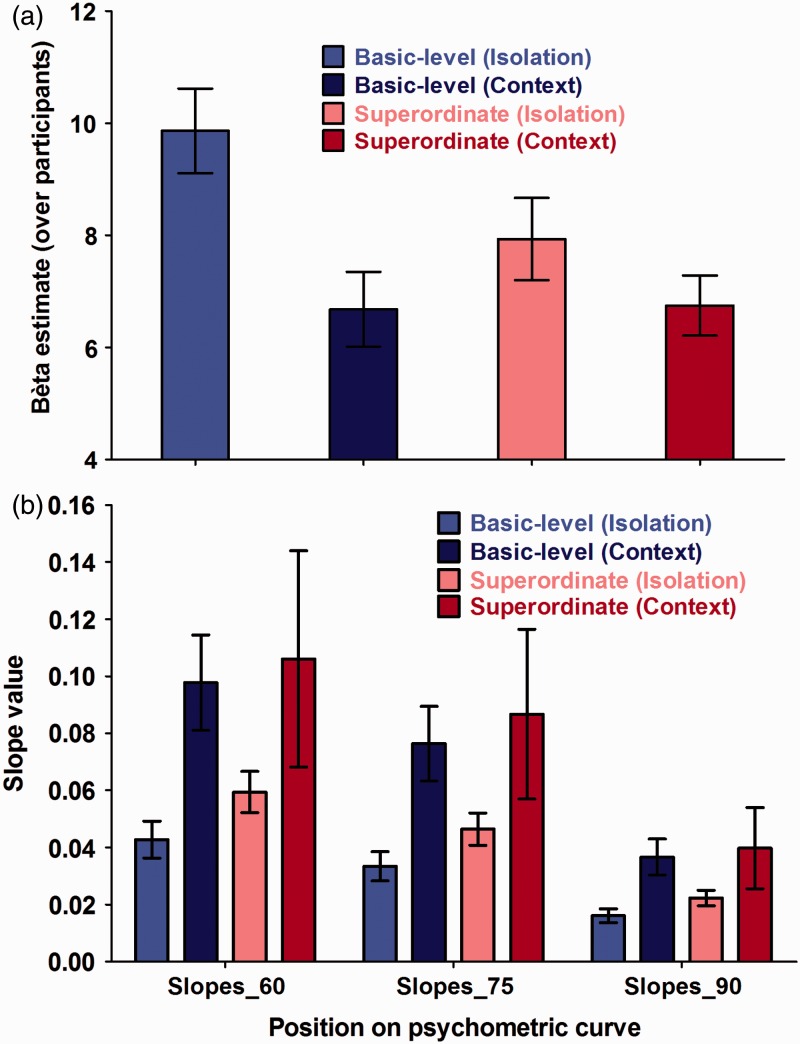
Overview of (a) average β estimates across participants and (b) average steepness of the psychometric curve at 60%, 75%, or 90% correct categorization performance when rapidly detecting a basic-level object-in-isolation (light blue bar), a basic-level object-in-context (dark blue bar), a superordinate-level object-in-isolation (pink bar), or a superordinate-level object-in-context (red bar). The data are represented as the mean performance across participants, with error bars depicting the standard error of the mean (*SEM*).

Hypothesis 2: Influence of object presentation mode on ultrarapid object detection (Figure 8).

**Table 1.** Overview of the Regression Parameter Estimates for the Main Parameters, Alpha (α) and Bèta (β), of the Individual Sigmoid Maximum Likelihood Fits.

Parameter	Estimate (*SE*)	*P*(χ^2^)	95% CI
Alpha (α)			
Intercept	28.13 (.92)	2.00 × 10^−16^	[26.33, 29.93]
Presentation mode	5.19 (1.19)	2.41 × 10^−5^	[2.86, 7.52]
Level of categorization	−1.88 (.66)	5.11 × 10^−3^	[−3.17, −.59]
Bèta (β)			
Intercept	8.91 (.49)	2.00 × 10^−16^	[8.42, 9.87]
Presentation mode	−2.19 (.67)	1.25 × 10^−3^	[−3.50, −.88]
Level of categorization	−.88 (.67)	1.90 × 10^−1^	[−2.19, .43]

*Note.* These provided an overall estimation of the time-dependent categorization performance in each test session per participant and were used separately as DV in a General Linear Modeling (GLMM) approach (McCullagh, 1984).

We found a significant main effect of the between-subjects variable presentation mode for α (*t*(143) = 4.37; *P*(χ^2^) = 2.41 × 10^−5^). This parameter provides an estimate of the overall PT necessary for participants to correctly judge whether a predefined object, either in context or in isolation, was presented or not. More precisely, the current results indicated that participants were generally faster in detecting an object-in-isolation than in detecting an object embedded within a meaningful context (Hypothesis 2). Simultaneously, we also found a significant main effect of presentation mode for β (*t*(272) = −3.26; *P*(χ^2^) = 1.25 × 10^−3^) in which participants were found to have steeper, PT-dependent, slope values in the context condition compared with the object-in-isolation condition. This difference in steepness was similar across the entire psychometric curve (60% slope: *t*(272) = 2.29; *P*(χ^2^) = .02 | 75% slope: *t*(272) = 2.39; *P*(χ^2^) = .02 | 90% slope: *t*(272.00) = 2.28; *P*(χ^2^) = .02). When translating this in terms of the specific threshold values, we found that participants only performed better in the object-in-isolation condition, compared with the context condition, at lower PT (60% threshold: *t*(141) = 3.66; *P*(χ^2^) < .001 | 75% threshold: *t*(140) = 2.28; *P*(χ^2^) = .03). When PT became larger, this difference in performance disappeared (90% threshold: *t*(138) = .25; *P*(χ^2^) = .80). When graphically exploring this effect further (Figures 6 and 7), we observed a general distribution shift for both threshold (α) and slope (β) values. More precisely, the objects-in-isolation distributions peak earlier than the objects-in-context distributions. See Table 1 for parameter estimates and 95% confidence intervals of the final models.

### Mask control experiment

Our analysis of the mask control experiment (see Appendix D for parameter estimates and 95% confidence intervals for the main parameters, α and β, of the individual sigmoid maximum likelihood fits) provided a clear replication of our experimental findings. More precisely, we found significant main effects of level of categorization (*t*(29) = −2.14; *P*(χ^2^) = .04) and presentation mode (*t*(29) = 7.47; *P*(χ^2^) < .01) for α and a main effect of presentation mode (*t*(29) = −2.13; *P*(χ^2^) = .04) for β. This indicated that the difference in mask type, used in either the object-in-isolation or the object-in-context condition, was no confounding variable in explaining the current results.

## Discussion

Hypothesis 1: Basic versus superordinate advantage in ultrarapid categorization.

We observed a consistent advantage across participants to rapidly identify superordinate-level, compared with basic-level, object information correctly (Hypothesis 1). This was in line with previous studies on ultrarapid categorization without perceptual masking (e.g., Poncet & Fabre-Thorpe, 2014; Praβ et al., 2014; Vanmarcke & Wagemans, 2015), and the predictions formulated based on the Leabra model (O’Reilly et al., 2013). This model stated that a predefined search task would allow top-down biasing of the relevant visual features even when stimulus PT lasted long enough to allow recurrent processing to influence the initial bottom-up sweep of information in the visual cortex (Bar, 2004; De Cesarei et al., 2015). More specifically, this idea follows from the PDP prediction that the categorization mechanism uses a general-to-specific process of conceptual differentiation, allowing unambiguous and well-learned object recognition with prolonged stimulus PT to occur in a dominantly bottom-up manner (Liu, Agam, Madsen, & Kreiman, 2009; VanRullen & Koch, 2003). The observed basic-level advantage in verbal semantic labeling tasks without any time constraints (Mervis & Rosch, 1981; Rosch et al., 1976) only follows when an active, conscious labeling of different semantic categories becomes necessary to resolve the given task (Rogers & Patterson, 2007). Simultaneously, Fabre-Thorpe and coworkers followed a similar reasoning claiming that the visual processing stage of object categorization has the property of the observed superordinate-level advantage, while active semantic processing leads to a basic-level advantage (Fabre-Thorpe, 2011; Joubert et al., 2008; Macé et al., 2009). They argued that the requirement for lexical access was critical: the behavioral superordinate- versus basic-level categorization advantage was determined by the extent to which the semantic domain canceled out the superordinate-level advantage in the visual domain. This prediction was supported by evidence indicating that ultrarapid categorization was color-blind (Delorme, Richard, & Fabre-Thorpe, 2010), robust to contrast reductions (Macé, Delorme, Richard, & Fabre-Thorpe, 2010) and relied on very coarse object representations (Thorpe, Gegenfurtner, Fabre-Thorpe, & Bülthoff, 2001). Furthermore, different studies indicated that rapid categorization can be performed in the near absence of attention (e.g., Li, VanRullen, Koch, & Perona, 2002; Rousselet, Macé, & Fabre-Thorpe, 2003). The detection of objects in ultrarapid categorization was therefore regarded as a preattentive and automatic process (VanRullen, Reddy, & Koch, 2004). Recurrent processing even was found to incur small costs in raw overall performance in relatively simple categorization tasks (O’Reilly, 2001). It was argued that these costs could provide a processing benefit in more complex, conscious recognition problems involving generalization or occlusion across nonvisual semantic dimensions. This argumentation would fit the predictions of the PDP theory within the more general cognitive framework of the reverse hierarchy theory of visual processing (Ahissar & Hochstein, 2004; Hochstein & Ahissar, 2002). Ultrarapid categorization can be successfully completed by the rapid and implicit bottom-up processing of visual information without gaining any processing advantage when activating explicit, attention-focused top-down or reverse hierarchical pathways to effectively inform low-level representations in the visual cortex.

Hypothesis 2: Influence of object presentation mode on ultrarapid object detection.

We predicted that participant performance would only be influenced by the congruent context when and if longer PT in perceptually masked rapid categorization allowed recurrent processing to affect response speed (Hypothesis 2). Results indicated that participants were generally faster in detecting an object-in-isolation correctly than in detecting an object embedded within a meaningful context, when stimulus PT remained short. When stimulus PT and categorization accuracy increased, differential performance between both conditions decreased rapidly and it disappeared completely with almost perfect categorization performance. These outcomes argue against a contextual processing advantage for participants who are instructed to rapidly detect a salient object in a masked ultrarapid categorization task. This would suggest that embedding the salient object within a meaningful surrounding initially increases stimulus ambiguity and complexity and therefore increases the overall task difficulty (O’Reilly, Wyatte, Herd, Mingus, & Jilk, 2013). This contradicted with previous findings, indicating the existence of a reaction times advantage when rapidly detecting objects-in-context, compared with objects-in-isolation, in an ultrarapid categorization task (e.g., Crouzet et al., 2012; Sun, Simon-Dack, Gordon, & Teder, 2011). The absence of such a contextual facilitation effect could be linked to the absence of perceptual masking in previous categorization designs (Fahrenfort et al., 2007). This prediction follows from the idea that masking derives its effectiveness from disrupting recurrent processing, while leaving feedforward signals intact (Lamme & Roelfsema, 2000). These recurrent connections have been suggested to play an integral role in a range of visual processes (Hochstein & Ahissar, 2002; Kastner & Ungerleider, 2000; Spratling & Johnson, 2004), such as figure-ground segmentation (Lamme et al., 2002). The latter process thereby seems especially important within the current time-dependent and masked ultrarapid categorization design. Due to the lack of contextual distractor elements or items in the isolated object condition, object identification is more accurate than the detection of objects embedded in a congruent background. We therefore predict that scene context will only facilitate response speed in ultrarapid categorization, when stimulus PT becomes larger than the time needed for participants to rapidly categorize the presented objects or scenes nearly perfectly (Davenport, 2007; Rémy et al., 2013). This contextual processing advantage might be based on excitatory recurrent processing, predominantly selecting the most likely object category within the contextual surrounding (Bar & Ullman, 1996; Fabre-Thorpe, 2011). More precisely, it was shown that humans can implicitly learn the temporal covariance of semantic categories of natural scenes (Brady & Oliva, 2008) and the global features of these scenes could be used to modulate the saliency of different contextual regions to guide visual search to pertinent scene locations (Torralba, Oliva, Castelhano, & Henderson, 2006). This would further underline the flexible dynamics of object categorization, depending jointly on the level of abstraction, time for perceptual encoding, and category context (Mack & Palmeri, 2015).

### Future Research

Because task performance depends on the intersection between task demand and object information, performance cannot be described in absolute terms (Schyns, 1998). Concretely, in our study, this means that performance depends on how much information is available to perform the task in each target image and in each nontarget image, on the similarities among images of each group, and also on the information overlap between target and nontarget images. So unless task-related information can be quantified for every image, it remains difficult to directly compare absolute performance between image categories and between tasks. For similar (and additional) reasons, differences in stimulus PT can also not directly, or indirectly, reflect the timing of the corresponding (underlying) brain processes (VanRullen, 2011). As a result, the estimation of processing speed for different image categories or in different tasks, and its generalization across different image sets, has to be done with caution. The more conservative conclusion, when two psychometric functions are found to differ, is that the two processes cannot be equated, and thus rely (at least in part) on distinct neuronal mechanisms. Future research on the influence of scene congruency and top-down processing during (ultrarapid) categorization should further focus on quantifying the low-level image properties (e.g., orientation, complexity, and shape) of the selected stimulus set (Joubert, Rousselet, Fabre-Thorpe, & Fize, 2009; Wichmann et al., 2006) and benchmarking it based on the available information in the specific images (VanRullen, 2011). This is necessary because the diversity of the image set by itself is no guarantee to avoid possible systematic differences between various image classes, and it has been shown that these differences can allow participants to discriminate between natural image categories almost perfectly (Brand & Johnson, 2014). For instance, it might be that different distributions of attention facilitate the extraction of different types of information within a scene (Brand & Johnson, 2014; Chong & Treisman, 2005). When attention is focused locally (e.g., on more low-level physical properties), features are bound together resulting in the identification of an object. When attention is distributed more globally (e.g., on more high-level physical properties), the semantic meaning of a scene is extracted based on its global layout. Finally, future research should also focus more on using electrophysiological, rather than psychophysical, methods to pinpoint the precise latency of the brain processes involved during categorization.

## Author's Note

Authors Steven Vanmarcke and Filip Calders contributed equally to this work.

## Appendix A

Overview of the amount of participants, included in the final analysis of the ultrarapid categorization task, in each of the different test conditions. No between-subject differences in performance were observed for the different detection tasks (e.g., car, plane,…) for either within-basic- (bed vs. chair vs. car vs. airplane) or within-superordinate- (furniture vs. vehicle) level categorization.

**Table:**

Number of participants in each condition?		Superordinate-level categorization			
		Furniture?		Vehicle?	
		Object-in- isolation	Object-in- context	Object-in- isolation	Object-in- context
Basic-level categorization	Bed?	18 Participants	18 Participants	–	–
	Chair?	17 Participants	14 Participants	–	–
	Car?	–	–	17 Participants	18 Participants
	Airplane?	–	–	16 Participants	18 Participants

### Appendix B

We used the alpha (α) and bèta (β) values, of each of the participants, as the dependent variables in a mixed ANOVA with presentation mode (object-in-context vs. object-in-isolation condition) as a between-subjects factor and level of categorization (basic vs. superordinate) as a within-subjects factor. Participants were regarded as a random factor.

**Table:**

Parameter	*F* statistic	*p*	Effect size (η^2^_p_)
Alpha			
Presentation mode	*F*(1, 134) = 18.96	2.60 × 10^−5^	η^2 ^= .12
Level of categorization	*F*(1, 134) = 7.59	7.00 × 10^−3^	η^2 ^= .05
Presentation Mode × Level of Categorization	*F*(1, 134) = .23	.63	η^2 ^= 2.00 × 10^−3^
Bèta			
Presentation mode	*F*(1, 134) = 9.74	2.00 × 10^−3^	η^2 ^= .07
Level of categorization	*F*(1, 134) = 1.97	.16	η^2 ^= .01
Presentation Mode × Level of Categorization	*F*(1, 134) = 2.58	.11	η^2 ^= .02

### Appendix C

Results of the modeling scheme—for both main psychometric parameters separately—in terms of fixed effects (with the *SD* between brackets) and goodness-of-fit. Based on a maximum likelihood estimation, we calculated the deviance values and selected the final model by evaluating the drop in deviance, together with the Akaike and Bayesian Information Criterion values. More precisely, we regarded the former as our main criterion for model selection, while the latter two information criterions provided extra information. The different models were always compared with the previous model and for alpha (α), Model C is the selected model with the best found fit (with presentation mode and level of categorization as main predictors), while in bèta (β) the best fit is observed in Model B (with presentation mode as main predictor).

**Table:**

Fixed effects	Model A	Model B	Model C	Model D	Model E
Alpha (α)					
Intercept	29.91 (.64)	27.20 (.87)	28.13 (.92)	27.96 (.99)	36.13 (11.80)
Presentation mode		5.15 (1.19)	5.19 (1.19)	5.50 (1.36)	5.59 (1.36)
Level of categorization			−1.88 (.66)	−1.55 (.96)	−1.55 (.96)
Presentation Mode × Level of Categorization				−.63 (1.33)	−.63 (1.33)
Age					−.47 (.59)
Gender					−11.62 (14.85)
Age × Gender					.66 (.77)
Goodness-of-fit					
Deviance	1881.4	1863.9	1856	1855.8	1854.5
Drop in Deviance	–	17.5	7.9	.2	1.3
Akaike Information Criterion	1887.4	1871.9	1866	1867.8	1872.5
Bayesian Information Criterion	1898.2	1886.3	1884	1889.4	1905.0
Bèta (β)					
Intercept	7.75 (.35)	8.91 (.49)	9.34 (.59)	9.86 (.68)	19.61 (6.53)
Presentation mode		−2.19 (.67)	−2.19 (.67)	−3.18 (.94)	−3.13 (.93)
Level of categorization			−0.88 (.67)	−1.93 (.97)	−1.93 (.96)
Presentation Mode × Level of Categorization				1.99 (1.34)	1.99 (1.32)
Age					−0.46 (.33)
Gender					−1.01 (8.21)
Age × Gender					−.13 (.42)
Goodness-of-fit					
Deviance	1713.7	1703.3	1701.6	1699.4	1691.7
Drop in Deviance	–	10.4	1.7	1.2	7.7
Akaike Information Criterion	1719.7	1711.3	1711.6	1711.4	1708.1
Bayesian Information Criterion	1730.5	1725.8	1729.6	1733.0	1733.3

### Appendix D

Overview of the regression parameter estimates for the main parameters, alpha (α) and bèta (β), of the individual sigmoid maximum likelihood fits of the mask control experiment. These provided an overall estimation of the time-dependent categorization performance in each test session per participant and were used separately as DV in a General Linear Modeling (GLMM) approach (McCullagh, 1984).

**Table:**

Parameter	Estimate (*SE*)	*P*(χ^2^)	95% CI
Alpha (α)			
Intercept	28.55 (1.74)	2.00 × 10^−16^	[25.14, 31.96]
Presentation mode	7.47 (2.20)	2.04 × 10^−3^	[3.16, 11.78]
Level of categorization	−3.55 (1.66)	4.13 × 10^−2^	[−6.80, −.30]
Bèta (β)			
Intercept	6.64 (.80)	5.90 × 10^−11^	[5.07, 8.21]
Presentation mode	−2.13 (.97)	3.66 × 10^−2^	[−4.03, −.23]
Level of categorization	.86 (.85)	3.20 × 10^−1^	[−.81, 2.53]
